# Prognostic Significance of Serum Free Light Chains in Chronic Lymphocytic Leukemia 

**DOI:** 10.1155/2013/359071

**Published:** 2013-10-29

**Authors:** Katerina Sarris, Dimitrios Maltezas, Efstathios Koulieris, Vassiliki Bartzis, Tatiana Tzenou, Sotirios Sachanas, Eftychia Nikolaou, Anna Efthymiou, Katerina Bitsani, Maria Dimou, Theodoros P. Vassilakopoulos, Marina Siakantaris, Maria K. Angelopoulou, Flora Kontopidou, Panagiotis Tsaftaridis, Nikolitsa Kafasi, Gerasimos A. Pangalis, Panayiotis P. Panayiotidis, Stephen Harding, Marie-Christine Kyrtsonis

**Affiliations:** ^1^Hematology Section of the First Department of Propedeutic Internal Medicine, Laikon University Hospital, Agiou Thoma 17, 11527 Athens, Greece; ^2^Immunology Department, Laikon General Hospital, Agiou Thoma 17, 11527 Athens, Greece; ^3^The Binding Site Ltd, B15 1QT, Birmingham, UK

## Abstract

*Background*. Serum free light chains (sFLC), the most commonly detected paraprotein in CLL, were recently proposed as useful tools for the prognostication of CLL patients. *Objective*. To investigate the prognostic implication of sFLC and the summated FLC-kappa plus FLC-lambda in a CLL patients' series. *Patients and Methods*. We studied 143 CLL patients of which 18 were symptomatic and needed treatment, while 37 became symptomatic during follow-up. Seventy-two percent, 18%, and 10% were in Binet stage A, B and C, respectively. Median patients' followup was 32 months (range 4–228). *Results*. Increased involved (restricted) sFLC (iFLC) was found in 42% of patients, while the summated FLC-kappa plus FLC-lambda was above 60 mg/dL in 14%. Increased sFLC values as well as those of summated FLC above 60 were related to shorter time to treatment (*P* = 0.0005 and *P* = 0.000003, resp.) and overall survival (*P* = 0.05 and *P* = 0.003, resp.). They also correlated with **β**2-microglobulin (*P* = 0.009 and *P* = 0.03, resp.), serum albumin (*P* = 0.009 for summated sFLC), hemoglobin (*P* < 0.001), abnormal LDH (*P* = 0.037 and *P* = 0.001, resp.), Binet stage (*P* < 0.05) and with the presence of beta symptoms (*P* = 0.004 for summated sFLC). *Conclusion*. We confirmed the prognostic significance of sFLC in CLL regarding both time to treatment and survival and showed their relationship with other parameters.

## 1. Introduction 

Chronic lymphocytic leukemia is the most common type of leukemia in the Western world accounting for 40% of all leukemias. It affects mainly elderly patients as the median age of diagnosis is about 72 years and the male to female ratio is 2 : 1. So far, Rai and Binet staging systems are used for predicting CLL patients' outcome. Other prognostic markers, which have been established but mainly concern symptomatic CLL patients, are lymphocyte CD38 expression, presence of ZAP-70, immunoglobulin (Ig) heavy gene mutation status, and cytogenetic profile [1–6]. In symptomatic patients the presence of unmutated Ig heavy chain variable region, the presence of ZAP-70, and CD-38 expression predict worse clinical outcome. Chromosomal abnormalities with importance for disease prognosis are deletion of long arm of chromosome 13, deletion of petit arm of chromosome 17 (del p17), and deletion of the long arm of chromosome 11 (del q11) with the first indicating a better prognosis than the last ones. Nevertheless the presence of these factors in patients does not signify that they should start treatment in the absence of symptomatic disease. 

Immunoglobulins (Igs) are produced by terminally differentiated B cells (either plasma cells or long lived memory cells), with the capacity to produce antibodies with high affinity for the immunizing antigen which are composed by 2 heavy and 2 light chains [7]. During this procedure a small excess amount of light chains are produced and released in the plasma/serum in the form of serum immunoglobulin free light chains (sFLC). It has recently been found that 38% of patients eventually developing CLL displayed abnormal sFLC ratio (FLCR) up to 10 years before CLL diagnosis and another 16% had polyclonal sFLC elevation in the same time frame preceding diagnosis [8]. 

Recent data have shown that serum free light chains and their ratio may constitute prognostic factors in CLL [9–15]. Serum free light assays have already been shown to improve detection, management, and prognostication in plasma cell dyscrasias [16–18]. In diffuse large B-cell lymphomas, increased levels of sFLC were shown to be an independent, adverse prognostic factor for overall survival (OS). Also abnormal sFLC ratio can help in CNS lymphomas diagnosis [19]. Recently abnormal sFLC ratio was found to play an important prognostic role in multiple myeloma [16], AL Amyloidosis [20], and Waldestrom's macroglobulinemia [17]. Moreover, abnormal FLCR is considered a risk factor for the progression of MGUS [21], solitary plasmacytoma [22], and smoldering myeloma [23] into multiple myeloma. 

It is of great importance to identify simple prognostic markers that could be widely applied in clinical practice and with low cost, in order to predict the group of asymptomatic patients that will shortly require therapy [24]. We therefore tested the eventual prognostic implication of sFLC in a cohort of CLL patients at diagnosis.

## 2. Patients and Methods 

### 2.1. Patients

Frozen sera from patients that fulfilled the 1996 CLL criteria [25] were drawn at diagnosis in 143 consecutive CLL patients with available sera. At presentation 18 (13%) of them needed immediate treatment, while all the others were asymptomatic and were only regularly followed. During followup 37 (26%) developed symptoms and required treatment administration. Seventy-two percent, 18%, and 10% of patients were in Binet stage A, B, and C respectively. 

Patients' standard workup at diagnosis included physical examination and whole body CT scanning for the evaluation of eventual lymph node swelling and organomegaly, complete blood counts and cell morphology evaluation on blood smears, bone marrow aspiration and biopsy, blood or marrow lymphocyte immunophenotype, and biochemical background including serum lactate dehydrogenase (LDH) and serum protein electrophoresis, while serum beta-2-microglobulin and fluorescent *in situ* hybridization (FISH) studies for del p17 and del q11, were tested in a subset of patients only (48% and 24%, resp.). 

Light chain restriction was established by flow cytometry or bone marrow biopsy immunohistochemistry. The patients' characteristics can be seen in Table 1; median patients' followup was 32 months (range 4–228). 

### 2.2. Methods

Serum free light chain values were retrospectively determined by nephelometry (Freelite^TM^, the Binding Site Birmingham, UK) in frozen sera drawn at diagnosis. 

Abnormal sFLC and FLCR values were defined as any values out of the 95th percent percentile normal ranges reported by the manufacturer [26, 27], meaning 3.3 to 19.4 mg/L for kappa free light chain, 5.7 to 26.3 mg/L for lambda free light chain, and 0.31 to 1.2 for FLCR. In this series, all patients but one had normal serum creatinine, so abnormal FLC values were assessed without adjustment for renal failure.

We evaluated the prognostic significance of the summated sFLC, using as cut off values (1) their median level, (2) the value of 60 mg/L proposed by Morabito et al. [15] that performed a receiver operating characteristics analysis in order to establish the most suitable sFLC (*κ* + *λ*) cutoff value and showed it was 60.6 mg/mL (area under the curve = 0.62; *P* < 0.0001), (3) we additionally tested arbitrarily a cutoff of 50 mg/L, because few early stage CLL patients had a summated FLC above 60 mg/L.

Statistical analysis was performed using SPSS v15.0. Correlations between disease variables derived from patients' standard workout at diagnosis and sFLC values were evaluated by the chi square test when assessed as categorical variables and by the Kendall's test if one of them was categorical. 

With regard to survival and time to first treatment (TFT), the prognostic significance of abnormal sFLC and ratios were determined by univariate Cox regression analysis. Kaplan Meier method was used for pictorial representation of survival and TFT. 

## 3. Results

### 3.1. sFLC Values in Patients

Sixty-nine percent of patients presented with kappa light chain restriction and 31% with lambda monoclonality. 

Kappa sFLC ranged from 2.54 to 196 mg/L (median 19.2 mg/L) and lambda from 9.19 to 121 mg/L (median 20 mg/L) in kappa-and lambda-restricted patients, respectively. Increased involved (restricted) sFLC (iFLC), either kappa or lambda, were found in 42% of patients. 

sFLC ratio (FLCR) ranged from 0.02 to 496 (median 0.95). Abnormal FLCR values, suggesting light chain paraprotein presence, were present in 30% of patients. 

The summated FLC-kappa plus FLC-lambda ranged from 9.4 to 217.4 mg/L (median 33,1 mg/L); it was higher than 60 mg/L in 14%.

### 3.2. Correlations between sFLC Values and Disease Markers

**Figure 1:**
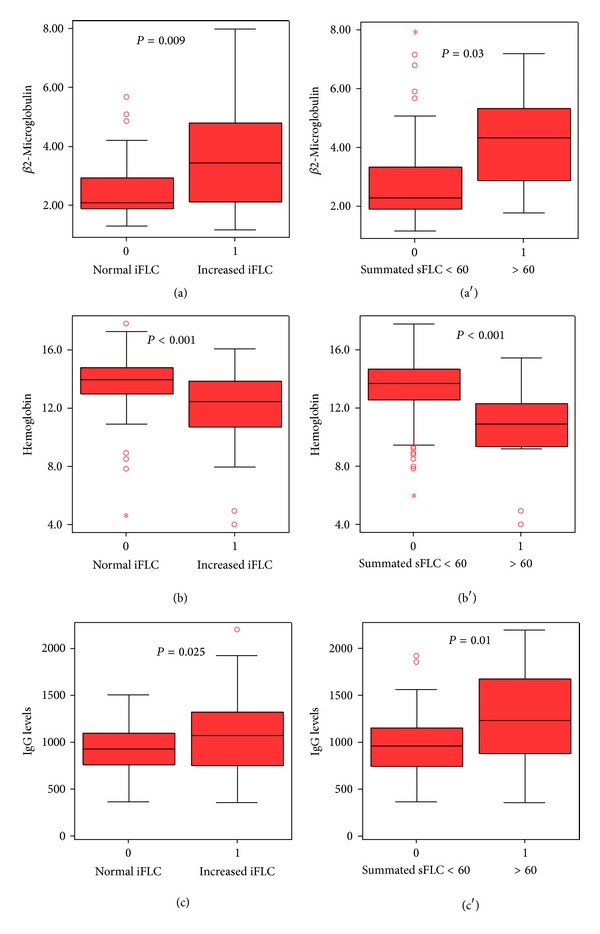


iFLC and summated sFLC kappa + lambda > 60 significantly correlated with Binet stage (*P* = 0.039 and *P* = 0.02, resp.), the presence of lymphadenopathy (*P* = 0.02 and 0.05, resp.), hemoglobin level (negatively), (*P* = 0.000003 and *P* = 0.0002, resp.), white blood cells counts (*P* < 0.01 both), lymphocyte absolute number (*P* < 0.01 both), serum immunoglobulin IgG level (*P* = 0.025 and 0.01, resp.), abnormal LDH (*P* = 0.037 and 0.001 resp.), beta-2-microglobulin (*P* = 0.009 and 0.03, resp.), serum albumin (*P* = 0.009 only for FLC kappa + lambda > 60), and with the presence of beta-symptoms (*P* = 0.004 only for summated sFLC > 60) (Figure 1). 

No correlations were found with the presence of spleen enlargement and CD38 expression while the number of cases tested for del p17, del q11, and IGVH status are too small to reach conclusions. 

FLCR did not show any significant correlation with disease variables.

### 3.3. Correlations between sFLC, FLCR, Summated sFLC Values and, Survival

#### 3.3.1. Time to First Treatment (TFT)

**Figure 2:**
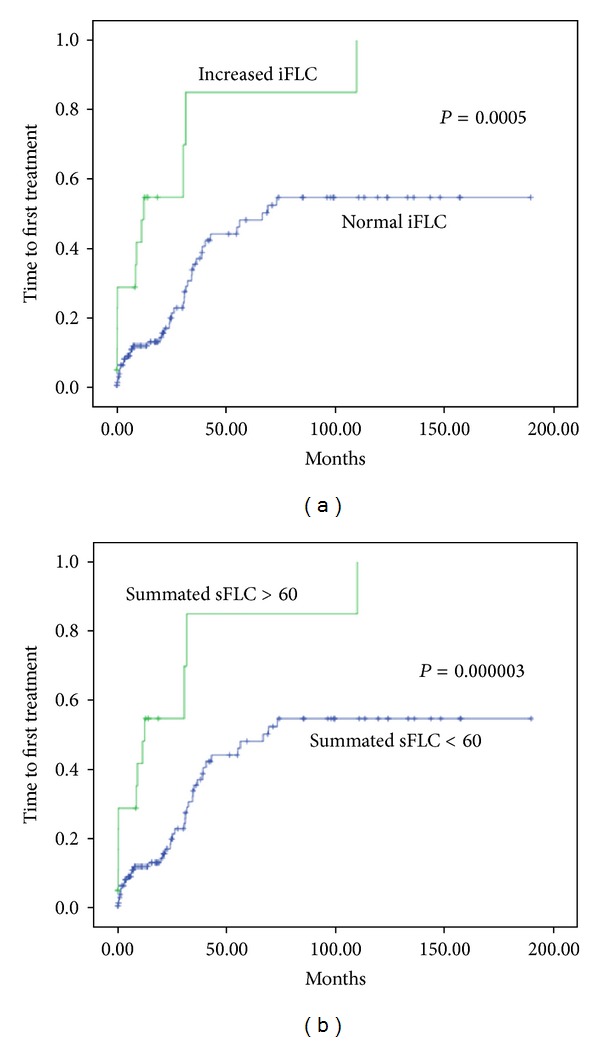


Increased involvment sFLC (iFLC) values as well as values of summated sFLC kappa + lambda > 60 were related to shorter TFT (*P* = 0.0005 and *P* = 0.000003, resp.) as shown in Figure 2, while neither values of summated FLC were above median nor was FLCR.

#### 3.3.2. Overall Survival (OS)

**Figure 3:**
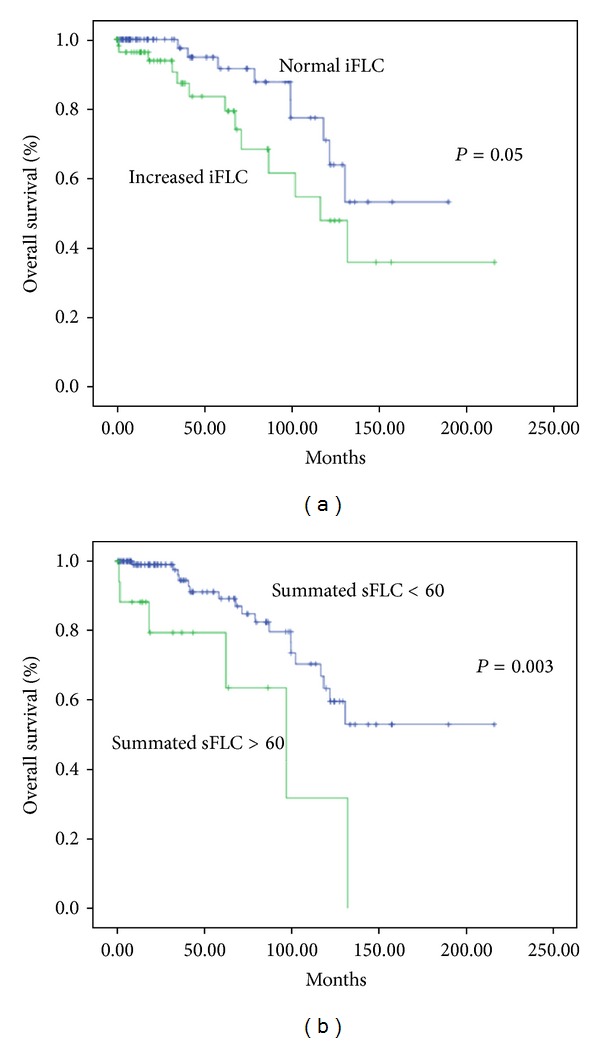


Increased iFLC and summated sFLC above 60 correlated with shorter OS (*P* = 0.05 and *P* = 0.003, resp.) as shown in Figure 3, while neither values of summated FLC above were median nor was FLCR.

### 3.4. Other Variables with Prognostic Importance on OS and TFT

We also evaluated the prognostic value of some other routine laboratory variable tested during diagnostic workup, namely, Binet stage, beta-2-microglobulin, serum albumin, and LDH. Hemoglobin levels, platelet counts, and lymphocyte doubling time were not assessed as they constitute, by definition, criteria to start treatment. IGVH status and FISH results for adverse genetic markers were not assessed, as they were available in only a minority of patients. 

Binet stage correlated with both TFT and OS (*P* < 0.001 for both). Abnormal (above normal upper limit) LDH correlated with OS (*P* = 0.001) but not with TFT.

Beta-2-microglobulin above 3.5 mg/L correlated with TFT (*P* < 0.001) but not with OS. Serum albumin correlated neither with TFT nor with OS.

However, because the number of patients is too small for a comprehensive modeling, prognostic models combining iFLC or summated FLC with other variables of adverse outcome were not assessed.

## 4. Discussion 

Although CLL is usually an indolent disease and may not require treatment for years, some patients can experience a much more aggressive disease and a shorter survival. The general rule is that symptomatic CLL patients need treatment immediately while the others should be regularly followed. Existing established clinical and genetic prognostic markers and staging systems apply very well to symptomatic patients but not always to asymptomatic ones that represent about 2/3 of all CLL patients. For these patients overall survival (OS) highly depends on the time to first treatment (TFT). 

The most frequent paraprotein produced in CLL is serum free light chain in almost up to 50% of the patients. Accordingly, we also found increased involved FLC in more than 40% of patients and an abnormal FLCR, thus, confirming the monoclonal nature of sFLC in 30% of cases. 

It has recently been shown that sFLC and their sum above 60.6 mg/L may contribute usefully to prognosis [15], mainly with regard to TFT. The first group that studied sFLC prognostic contribution in CLL was the one of Pratt et al. [10]. Using a Cox regression analysis, they identified 4 independent prognostic variables for overall survival, namely, Zap-70, *β*2-microglobulin, M-IgVH, and abnormal sFLC ratio. Patients with CLL with an abnormal sFLC ratio were significantly more likely to have U-IgVH, a Zap-70 positivity, a lymphocyte doubling time less than 12 months, and a high *β*2-microglobulin. In a similar way, in a study involving 84 patients with CLL, Perdigao et al. [13] showed a correlation among abnormal sFLCr, TFT, IgVH mutational status, and survival. In their study of 34 patients with CLL in various stages (median age, 66 years; male-female ratio of 1.9 : 1; median time from diagnosis, 41.5 months), Ruchlemer et al. found [28] an abnormal sFLCr in 53% of cases, which mostly correlated with advanced disease stage and increased *κ* chain. In a separate group of 120 patients with CLL (serum samples collected before initiation or 6 months after cessation of treatment), 71 patients (59%) had abnormal FLCR. In addition to improving M-protein detection in CLL, it was shown that abnormal low FLCR (indicating lambda FLC involvement) was associated with worse outcome. In addition, no correlation was found between sFLC and other prognostic factors (ZAP-70, CD38, cytogenetic markers, and Binet stage) implying, according to the authors, that sFLC is an independent prognostic factor in patients with CLL [9]. Unlike these results, we found a correlation between iFLC or the summated sFLC and Binet stage.

In their recent study, Maurer et al. [14] classified 339 newly diagnosed patients with CLL into 3 types: type 1 with elevated *κ* or *λ* FLC and abnormal sFLCr (monoclonal, *n* = 57 patients), type 2 with elevated kappa or lambda FLC and normal sFLCr (polyclonal, *n* = 52 patients), and type 3 with normal range kappa and lambda FLC and abnormal FLCR (monoclonal, *n* = 54 patients). Patients were followedup for a median of 47 months. Forty-nine percent of patients with sFLC abnormalities had a worse TFT and overall survival than those with normal sFLC. The authors concluded that sFLC is an important prognostic factor and is maintained after adjustment for Rai staging, with different types of sFLC abnormality affecting prognosis to various degrees. 

In 2010, Yegin et al. [11] in their retrospective study assessed the prognostic value of sFLC levels and FLCR in a cohort of 101 patients with CLL (median age, 62 years; male-female ratio, 68 : 33) followedup for a median of 29 (range, 1.1–234) months. sFLC levels were found to be high in 55 patients (54.5%), with 30 patients (29.7%) having abnormal FLCR, in agreement with our results. Median TFT was shorter in patients with high sFLC levels but not in low-risk patients with CLL. In addition, median TFT was not statistically different between patients with normal and abnormal FLCR. The median overall survival was shorter in patients with both high sFLC levels and abnormal FLCR, but this did not remain valid in the multivariate analysis, probably because of the small sample. Furthermore, patients with high sFLC levels and abnormal FLCR expressed higher CD38 levels and positivity, thus, indicating that these biomarkers are involved in stimulation of B-cell receptor on CLL proliferating cells. However, we did not find any correlation with CD38 expression. 

The finding that increased polyclonal sFLC also constituted an adverse marker for time to first treatment (TFT) in CLL was firstly reported by Maurer et al. [14] and almost immediately confirmed by Morabito et al. [15] that evaluated the sum of absolute kappa and lambda sFLC and found that the prognostic impact of the summated sFLC (kappa + lambda) value above 60.6 mg/L was a superior prognosticator of TFT than FLCR [15]. 

In our study, increased sFLC were found in 42% of the patients, while the summated FLC-kappa plus FLC-lambda was higher than 60 mg/dL in 14%. Increased involvement of sFLC (iFLC) values and values of summated sFLC above 60 were related to shorter TFT and OS, in agreement with the aforementioned publications; we additionally tested the prognostic potential of the summated FLC using as cutoff the median value and confirmed that the value of 60 mg/L was a better cutoff. We also found significant correlations with disease variables. Likewise, iFLC and/or summated sFLC above 60 correlated with *β*2-microglobulin, serum albumin, negatively with hemoglobin, white blood cell and lymphocyte counts, abnormal LDH, Rai and Binet stage, and with the presence of lymphadenopathy and beta-symptoms. The relationship with IgG is very interesting and its significance remains to be fully evaluated. The observed correlations of sFLC with *β*2-microglobulin, albumin, and IgG are reported here for the first time and their interpretation remains to be further studied. 

In the present study, Binet stage was, as expected, of prognostic relevance with regard to TFT and OS, while abnormal serum LDH was predictive of a worse outcome and increased beta-2-microglobulin of a shorter TFT. However, the number of patients is too small to build prognostic models associating iFLC or summated FLC with other variables of adverse outcome. This should be done in larger series.

## 5. Conclusion

The results of our study confirmed the significance of sFLC in CLL with regard to TFT and OS, showed their relationship with adverse prognostic clinical and laboratory parameters and suggested, in accordance with others, that these tests should be included in CLL patients' initial workout, as they offer additional prognostic information. 

## Figures and Tables

**Table 1:** Patients' characteristics.

Number of patients	143
Median age	63 years (range 37–87)
Sex male/female	64/79 (45%/55%)
Rai stage	
0	64 (45%)
I	46 (32%)
II	14 (10%)
III	14 (10%)
IV	5 (3%)
Binet stage	
A	89 (62%)
B	31 (22%)
C	23 (16%)
Lymphadenopathy	79 (55%)
Splenomegaly	23 (16%)
LDH (>normal upper limit)	20 (14%)
Hemoglobin, g/dL, median (range)	13,5 (4–17,8)
White blood cell counts, ×10^9^/L, median (range)	21,600 (7–600)
Lymphocytes absolute value, ×10^9^/L, median (range)	14 (5–580)
Beta-2-microglobulin, mg/L median (range)*	2,5 (1,18–8,8)
CD38 expression > 20%	14 (10%)
FISH and IGVH status**	34 (24%)
Symptomatic	18 (12,5%)
Median followup	32 months (range 4–228)

*Evaluated in 69 patients.

**The percentage of patients that were positive is too small to be evaluated.

## Abbreviations

sFLC: Serum free light chains
iFLC: Involved serum free light chains
FLCR: Free light chain ratio
TFT: Time to first treatment
OS: Overall survival
CLL: Chronic lymphocytic leukemia
IGVH Ig: Heavy chain variable region.
